# Mechanical and Physical Properties of an Experimental Chemically and Green-Nano Improved Dental Alginate after Proven Antimicrobial Potentials

**DOI:** 10.3390/gels9050429

**Published:** 2023-05-21

**Authors:** Lamia Singer, Christoph Bourauel

**Affiliations:** 1 Oral Technology, Dental School, Medical Faculty, University Hospital Bonn, 53111 Bonn, Germany; bourauel@uni-bonn.de; 2 Department of Orthodontics, Dental School, Medical Faculty, University Hospital Bonn, 53111 Bonn, Germany

**Keywords:** irreversible hydrocolloid gels, *Boswellia sacra*, tear strength, elastic recovery, detail reproduction

## Abstract

Objectives: Impression materials could be a source of cross-contamination due to the presence of microorganisms from blood and saliva inside the oral cavity. Nevertheless, routinely performed post-setting disinfection could compromise the dimensional accuracy and other mechanical properties of alginates. Thus, this study aimed to evaluate detail reproduction, dimensional accuracy, tear strength, and elastic recovery of new experimentally prepared self-disinfecting dental alginates. Methods: Two antimicrobial-modified dental alginate groups were prepared by mixing alginate powder with 0.2% silver nitrate (AgNO_3_ group) and a 0.2% chlorohexidine solution (CHX group) instead of pure water. Moreover, a third modified group was examined by the extraction of *Boswellia sacra* (*BS*) oleoresin using water. The extract was used to reduce silver nitrate to form silver nanoparticles (AgNPs), and the mixture was used as well in dental alginate preparation (*BS* + AgNP group). Dimensional accuracy and detail reproduction were examined as per the ISO 1563 standard guidelines. Specimens were prepared using a metallic mold engraved with three parallel vertical lines 20, 50, and 75 µm wide. Detail reproduction was evaluated by checking the reproducibility of the 50 µm line using a light microscope. Dimensional accuracy was assessed by measuring the change in length between defined reference points. Elastic recovery was measured according to ISO 1563:1990, in which specimens were gradually loaded and then the load was released to allow for recovery from the deformation. Tear strength was evaluated using a material testing machine until failure at a crosshead speed of 500 mm/min. Results: The recorded dimensional changes between all tested groups were insignificantly different and within the reported acceptable values (between 0.037–0.067 mm). For tear strength, there were statistically significant differences between all tested groups. Groups modified with CHX (1.17 ± 0.26 N/mm) and *BS* + AgNPs (1.11 ± 0.24 N/mm) showed higher tear strength values compared to the control (0.86 ± 0.23 N/mm) but were insignificant from AgNO_3_ (0.94 ± 0.17 N/mm). All tested groups showed elastic recovery values that met both the ISO standard and ADA specifications for elastic impression materials and tear strength values within the acceptable documented ranges. Discussion: The CHX, silver nitrate, and green-synthesized silver nanoparticles could be promising, inexpensive alternatives for the preparation of a self-disinfecting alginate impression material without affecting its performance. Green synthesis of metal nanoparticles could be a very safe, efficient, and nontoxic method, with the advantage of having a synergistic effect between metal ions and active chemical constituents of plant extracts.

## 1. Introduction

Alginates were originally developed in the 1930s, and they are among the regularly used dental materials in every dental practice for making diagnostic casts and digital models [1]. Alginates are available in the form of powder, which undergoes polymerization and gelation when mixed with water to turn into a gel and then elastic material. This process of transformation of the powder to gel is employed to make a replica of the teeth and soft tissues in dental clinics. Alginates have the advantages of being inexpensive, easy to use, of hydrophilic nature, and acceptable by patients [2]. The powder is formed of soluble sodium alginates, filler particles (diatomaceous earth), a calcium sulfate reactor, fluoride as an accelerator, and sodium phosphate as a retarder [3].

Although dental alginates are not as good as elastomers in the reproduction of surface details and dimensional stability due to imbibition and syneresis, the optimization of these properties is still required to be able to serve its purpose. The dimensional accuracy of an impression material directly affects the fit and retention of the indirect restoration, and consequently the final clinical outcome. Moreover, precise surface detail reproduction is very crucial in the construction process of working casts and virtual replicas, and, therefore, significantly detailed final restoration [4,5].

Mechanical properties of alginate such as elastic recovery and tear strength can define the quality of the impression and the final restoration [6]. Elastic recovery is the ability of an elastic material to return to its original shape after being deformed inside the mouth with negligible permeant deformation. The higher the elastic recovery of a material, the more precise the final restoration will be [7]. Moreover, tear energy is very important, especially in taking impressions in thin areas. High tear energy accounts for the high resistance of the impression to rupture and distortion, especially in areas with existing undercuts [8].

On the other hand, dental impressions and gypsum casts poured against them are considered sources of cross-contamination from saliva and blood inside the oral cavity. Sterilization by heat cannot be performed in cases of alginate due to its nature; therefore, only cold chemical disinfection can be applied [9]. The ideal disinfection technique must not negatively affect the physical and mechanical properties of the material and gypsum to produce an accurate final appliance [10]. Commonly used chemical disinfectants include alcohols, aldehydes, sodium hypochlorite, iodide compounds, and quaternary ammonium salts [11].

Spraying and immersion techniques are the two mainly used cold methods for the disinfection of alginate. However, these techniques present several shortcomings, as spraying provides only a surface decontamination effect, whereas immersion is not optimum for alginate due to its hydrophilic nature [10,11]. For hydrocolloids, the time and method of application of a disinfectant depend on the ability of the material to withstand the procedure without negatively affecting its properties [12]. On the other hand, if the disinfectant concentration, PH, or contact time is not enough in spraying or immersion, the effectiveness of disinfection will be compromised, particularly for hydrocolloids, where bacteria and viruses can penetrate through the porous structure and grow inside the impression [10,13].

A new approach of incorporating chemical antiseptics into the alginate powder or mixing water has shown to be effective in eradicating pathogens, without adversely affecting the impression properties [14,15]. Moreover, it could be postulated that the impregnation of decontaminators into the impression would lead to disinfection throughout the material and not just superficially as in conventional techniques [16,17].

Chlorhexidine (CHX) has profound antibacterial and antifungal potentials with great efficacy even at low concentrations. CHX can affect both aerobic and anaerobic bacteria and even destroy DNA and RNA viruses [18]. Furthermore, silver nitrate is a common silver salt, which is widely used in medicine due to its antibacterial properties. Silver nitrate efficacy could be related to the ability of silver ions to bind to the bacterial cell wall and DNA, causing bacterial inactivation and inhibition of their replication [19]. Recently, using plant extracts for the green synthesis of silver nanoparticles (AgNPs) is attracting high attention due to their role in reducing and stabilizing nanoparticles, in addition to being environmentally friendly and with enhanced therapeutic potentials [20]. Currently, green protocols for the synthesis of AgNPs by plants or microorganisms are evolving rapidly as a novel field of science that is known as green nanotechnology [21].

Disinfection of dental impressions has been a subject of interest and concern for many years. The main requirement for a disinfectant agent or technique is to be efficient in eliminating pathogens without negatively affecting the properties of the impression. The challenges associated with disinfecting irreversible hydrocolloids due to imbibition and syneresis have raised our interest in developing self-disinfecting dental alginate by mixing the powder with 0.2% CHX and 0.2% AgNO_3_ (CHX group and AgNO_3_ group). In addition, a *Boswellia sacra* plant extract was used in the green synthesis of AgNPs, and the mixture was used as well for the preparation of a third modified alginate group.

In our former study, UV-visible (UV–vis) spectroscopy, scanning electron microscopy (SEM), and energy-dispersive X-ray analysis (EDX) confirmed the biosynthesis of AgNPs using the *Boswellia sacra* extract in a simple, inexpensive, and ecologically friendly way. The chemical analysis of the *Boswellia sacra* (*BS*) extract revealed the presence of 41 different organic compounds that acted as reducing and stabilizing agents for the green synthesis of AgNPs. Moreover, the groups modified with CHX, AgNO_3_, and the *BS* + AgNPs showed significantly enhanced antimicrobial activity compared to the control against *Candida albicans* (*C. albicans*), *Streptococcus mutans* (*S. mutans*), *Escherichia coli* (*E. coli*), methicillin-resistant Staphylococcus aureus (MRSA), *Staphylococcus aureus* (*S. aureus*), and *Micrococcus luteus* (*M. luteus*)*,* whereas the *BS* + AgNPs and CHX groups were significantly different against almost all strains, in which the CHX-modified alginate reported significantly higher results, except for with MRSA and *E. coli* [22,23].

Therefore, based on these findings and the fact that the green synthesis of metal nanoparticles using the *Boswellia sacra* extract showed promising synergistic antimicrobial effects between metal ions and the phytotherapeutic agents of the plant extract, this study aimed to assess the impact of these self-disinfection modifications of alginate on detail reproduction, dimensional changes, tear strength, and elastic recovery.

## 2. Results and Discussion

### 2.1. Results

#### 2.1.1. Detail Reproduction

Only the 50 and 75 µm lines were reproduced in all tested groups, while the 20 µm line did not appear in almost all specimens. As per the ISO 1563 criterion, which assesses the ability of alginate impression material to reproduce the entire length of the 50 μm line, all the specimens passed the test (100% score 1, 0% score 0).

#### 2.1.2. Dimensional Accuracy

**Figure 1:**
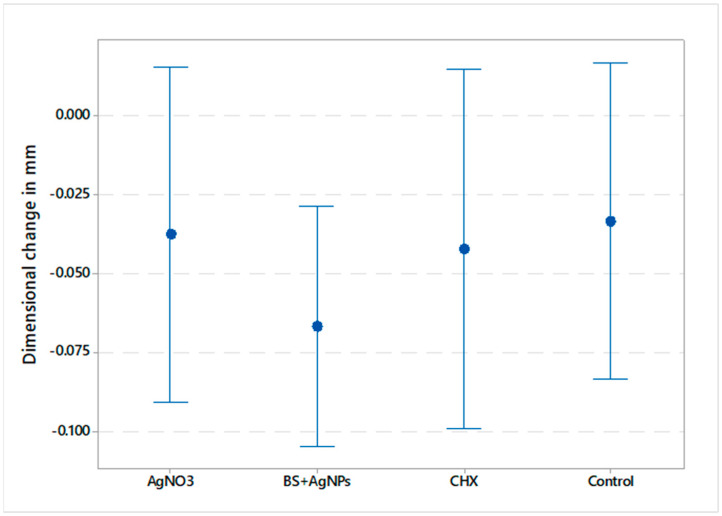
Interval plot illustrating the mean dimensional changes in millimeters in the vertical dimension and 95% confidence interval of all tested groups.

**Figure 2:**
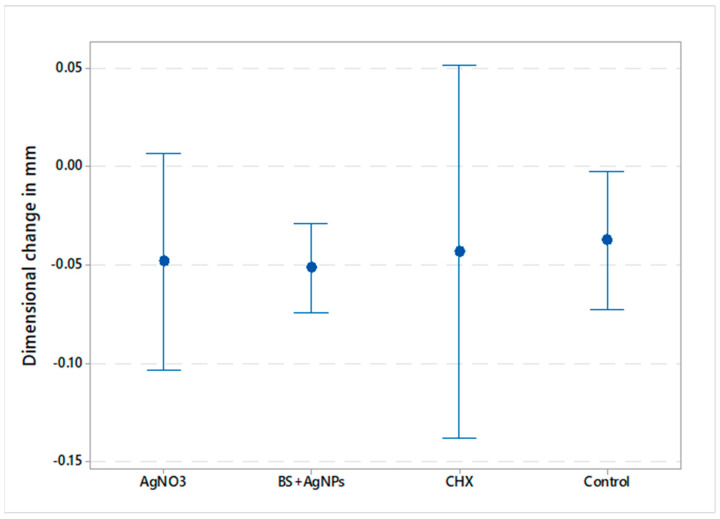
Interval plot illustrating the mean dimensional changes in millimeters in the horizontal dimension and 95% confidence interval of all tested groups.

Data showed parametric distribution, and thus, the mean and standard deviation values of the dimensional changes of the tested materials are represented in Figure 1 and Figure 2. The results indicated that the mean dimensional changes in mm were −0.038 ± 0.051 (AgNO_3_), −0.067 ± 0.036 (*BS* + AgNPs), −0.042 ± 0.046 (CHX), and −0.033 ± 0.054 (control) for the vertical dimension, whereas the mean values in the horizontal dimension were −0.048 ± 0.052, −0.051 ± 0.022, −0.043 ± 0.077, and −0.037 ± 0.038 for AgNO_3_, *BS* + AgNPs, CHX, and control, respectively. The results showed a statistically non-significant difference in dimensional changes between the four tested materials in the X (*p*-value = 0.619) and Y (*p*-value = 0.958) axes.

#### 2.1.3. Tear Strength

**Figure 3:**
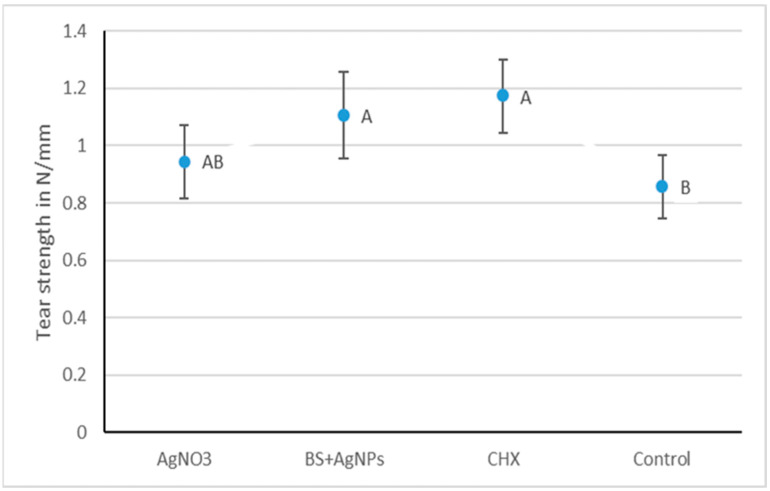
Interval plot representing the mean tear strength (N/mm) values and 95% confidence interval of all tested groups. Groups that do not share a letter are significantly different.

One-way ANOVA followed by Tukey’s post hoc test for pairwise comparison was used to compare the three different groups. Tear strength means and standard deviation values in N/mm are illustrated in Figure 3. The results revealed that there was a statistically significant difference between the tear strength of the tested materials (*p*-value = 0.002). *Bs +* AgNPs, AgNO_3_, and CHX showed comparable and significantly highest mean tear strengths of 1.11 ± 0.24 N/mm, 0.94 ± 0.17 N/mm, and 1.17 ± 0.26 N/mm, respectively. The control (0.86 ± 0.23 N/mm) was insignificantly different from AgNO_3_ but was significantly lower than CHX and *BS* + AgNPs.

#### 2.1.4. Elastic Recovery

**Figure 4:**
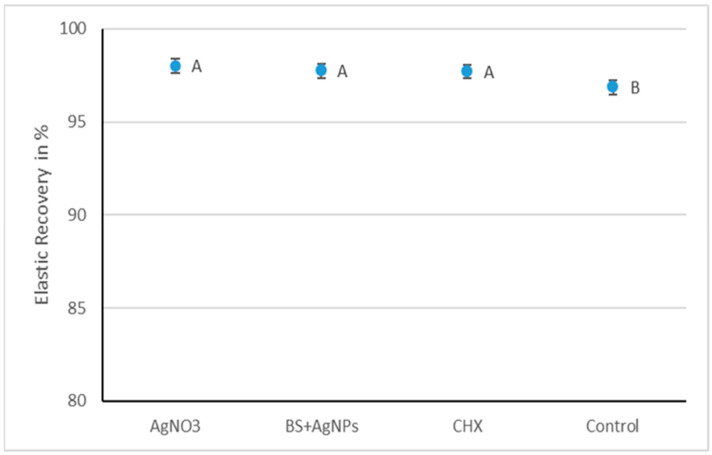
Interval plot representing the mean elastic recovery in % and 95% confidence interval of all tested groups. Groups that do not share a letter are significantly different.

The means and standard deviations of percentage recovery from deformation are represented in Figure 4. A one-way analysis of variance (ANOVA) indicated that there was a statistically significant difference in the elastic recovery of the four tested groups (*p*-value = 0.001). The AgNO_3_ group had a mean elastic recovery of 98.0% ± 0.3, which was insignificantly different from the *BS* + AgNPs (97.8% ± 0.8) and CHX (97.7% ± 0.7). The three modified groups were significantly higher than the control (96.9% ± 0.5).

### 2.2. Discussion

The present study was conducted on alginate hydrocolloid impression material, as it is a widely used dental material due to its attractive properties, such as acceptable detail reproduction, ease of manipulation, and low cost [1]. Disinfection of alginate impressions is a mandatory step to cease the spread of infectious diseases. Spraying and immersion techniques currently used for the disinfection of impression materials are far from ideal in alginate cases. Therefore, in the present study, some properties of alginate mixed with different disinfectant liquids instead of pure water were tested (CHX, AgNO_3_, and *Boswellia sacra* extract mixed with green-synthesized silver nanoparticles).

The antimicrobial activity of the modified groups was tested in our previous study using agar diffusion assays against *S. aureus*, methicillin-sensitive and resistant; *S. mutans; M. luteus* (four Gram-positive strains); one Gram-negative bacterium (*E. coli*); and a yeast (*C. albicans*). The results showed that CHX, AgNO_3_, and *BS* + AgNP groups were significantly more active than the control group against all tested strains. On the other hand, the antimicrobial activity of *BS* + AgNPs was comparable to the CHX group against *C. albicans* and MRSA. In addition, CHX was significantly more active compared to the other tested groups against *S. mutans*, *S. aureus*, *E. coli*, and *M. luteus*. Therefore, this study aimed to understand the impact of this new approach on the physical and mechanical properties after already exhibiting a profound antimicrobial efficacy [23].

Impression materials should have sufficient flow during impression taking to precisely replicate details of soft and hard oral tissue; consequently, it is crucial to have an optimum low viscosity [24]. The results of detail reproduction showed that all the specimens met the ISO requirement of recording the entire length of the 50 μm line. This may be attributed to the sufficient flow and the non-altered viscosity of the alginate impression before gelation even with the use of different mixing solutions other than pure water [24]. The results were in agreement with Omidkhoda et al. [25], who showed that adding silver nanoparticles to alginate for self-disinfection did not compromise the surface detail reproduction and flow properties.

The American Dental Association’s specification number 18 and the International Organization for Standardization’s 1563:1990, which are concerned explicitly with dental alginate, have neither specific requirements nor any limits on dimensional change values [26,27]. Dimensional accuracy measurements of alginate can be performed using several methods, including digital calipers, micrometers, dial gauges, and microscopes [28]. In this study, an optical microscope was used due to its high precision (0.0005 mm) to calculate the extent of dimensional changes after mixing alginate powder with three different antiseptic solutions (0.2% CHX, 0.2% AgNO_3_, and *BS* + AgNPs).

It has been reported that a range between 0.027- and 0.083-mm marginal discrepancy and/or a maximum value of 0.050 mm for a single unit is considered clinically acceptable for the fabrication of most indirect restorations [29,30]. Auspiciously, the results obtained in the vertical (between 0.038 and 0.067 mm) and horizontal dimensions (between 0.048 and 0.051 mm) for all tested groups were within the reported values and with no significant differences among all tested groups. This is in agreement with Mathew et al. [31] and Ismail et al. [32], who modified alginate material with hydrogen peroxide and povidone (PVP) iodine powder and found no changes in the dimensional accuracy of the tested groups.

The dimensional deviation between the metallic mold and alginate specimens was almost insignificantly different between all groups in the X and Y axes. The results obtained were negative for all the tested groups in the vertical and horizontal dimensions. Based on the findings, it could be hypothesized that CHX, Ag, and AgNPs consumed part of the mixing water ratio, which is crucial for the dissolution of the calcium sulfate reactor [33]. Dissolution of this reactor is important for the release of calcium ions that replace either the sodium or the potassium ions of the alginate to form an insoluble calcium alginate gel [34,35]. It was found that a reduction in the concentration of calcium ions increases the swelling capability of alginate beads and consequently causes the expansion of the material [36].

The tear strength of dental alginate is very important to be considered in areas with undercuts or insufficient thickness to resist tearing [37]. There is no defined protocol or specified values for the tear strength of impression materials in the ISO standard 1563 [27] or specification no. 18 of ANSI/ADA [26,38,39]. Tear strength evaluation in this study was performed using V-shaped specimens with a thickness of 4 mm to be similar to the clinically recommended thickness of the alginate impressions (4 to 6 mm range) [40].

The results of all the tested groups were within the acceptable documented range in the literature, which varies from 0.4 to 1.2 N/mm [38,40,41]. *BS +* AgNPs, AgNO_3,_ and CHX showed comparable and significantly highest mean tear strengths of 1.11 ± 0.24 N/mm, 0.94 ± 0.17 N/mm, and 1.17 ± 0.26 N/mm, respectively. The control (0.86 ± 0.23 N/mm) was insignificantly different from AgNO_3_ but was significantly lower than the other two modified groups. The elastic recovery of all tested groups (control 96.9% ± 0.5, *BS* + AgNPs 97.8% ± 0.8, CHX 97.7% ± 0.7, and AgNO_3_ 98% ± 0.3) met ISO 4823 [42] (≥96.5%) for elastomeric impression materials and ANSI/ADA specification no. 18-1992 (≥95%) [26] for hydrocolloid impression materials. The three modified groups recorded significantly improved elastic recovery values compared to the control.

The significantly higher elastic recovery and improved tear strength could be due to the presence of CHX ions, Ag ions, and silver nanoparticles that may have acted as filler particles and improved the mechanical properties. It has been hypothesized that tear strength and elastic recovery could be affected by certain factors, including the degree of cross-linking of the set alginate [43]. Moreover, a slight reduction in water amount could result in a material with a strong matrix and more tear resistance. This may be due to the role of water as a plasticizer and the presence of excess ions that can cross-link alginic chains more effectively; as a result, the final strength and elastic modulus could be improved while the setting time could be shortened [44].

This is in agreement with Fayez et al. 2016 [39] and Zarb et al. [45], who stated that any modifications of the given powder–liquid ratio, mixing technique, and filler content could result in alterations in the properties of the gel, tear energy, and elastic recovery. Several researchers also investigated the antimicrobial efficacy of metal oxide nanoparticles incorporated into alginate impression materials. They reported that these nanoparticles could be considered effective self-disinfecting agents for alginate impression materials with no adverse effect on the physical and mechanical properties [46,47].

One of the limitations of this study was that the impressions were taken with a standardized stainless-steel mold that does not fully resemble the behavior of the oral tissues, with regard to fluid absorption and the intrinsic free energy of teeth and oral soft tissues. Moreover, a future setup to examine the ability of alginate to flow and record fine details in the presence of saliva is currently being designed.

## 3. Conclusions

Within the framework of our former work and this study, CHX, AgNO_3,_ and *BS* + AgNPs could be considered promising additives for effective self-disinfecting alginate without compromising its physical and mechanical properties. Detail reproduction and accuracy of alginate were not negatively impacted by the different self-disinfection modifications. Elastic recovery was improved by the addition of CHX, AgNO_3,_ and *BS* + AgNPs. In addition, all groups were within the acceptable range for tear strength, with CHX and *BS* + AgNPs showing significantly higher tear strength values compared to the control group. Future work involving different concentrations of several metal ions and *B. sacra* is planned with testing their effect on the stability, setting time, hydrophilicity, and flow of alginate.

## 4. Materials and Methods

### 4.1. Materials

Conventional fast-set alginate (Pluradent GmbH and Co., Bornheim, Germany), superior Hojari Frankincense, *Boswellia sacra* gum (Dohfar mountains, Oman; imported by Jeomra Verlag, Georg Huber, Germany), silver nitrate ≥ 99.0% (Sigma-Aldrich, St. Louis, MO, USA, 209139-25G), and chlorohexidine (Caymen Chemical, Biomol GmbH, Hamburg, Germany) were used.

### 4.2. Methods

Control dental alginate and three modified antiseptic solutions were prepared to be used for mixing alginate instead of normal distilled water as follows:

***BS* + AgNP group:***Boswellia sacra* (*BS*) resin was washed, dried, and frozen at −16 °C overnight to facilitate the grinding of the resin into a fine powder without being softened inside the blender. Afterward, the powder was soaked in distilled water for 3 days, filtered using Whatman’s paper 1, and stored at 4 °C until usage. For preparation and reduction of the silver nanoparticles (AgNPs), a given amount of the above extract was added to 60 mL of 0.2% AgNO_3_ solution and stirred at a speed of 800 rpm. The mixture was incubated for 3 days at room temperature in the darkness with the solution color turning from white to dark brown, signifying the formation of AgNPs.

**0.2% CHX group:** An amount of 2 gm of chlorohexidine powder was added to 1000 mL distilled water and vortexed for 15 min until complete dissolution of the powder in the water.

**0.2% AgNO_3_ group:** An amount of 2 gm of 99% silver nitrate powder was added to 1000 mL of distilled water and mixed using a vortex mixer for several minutes.

**Control group:** Pure distilled water was used for alginate preparation without any additives.

#### 4.2.1. Detail Reproduction and Dimensional Accuracy

**Figure 5:**
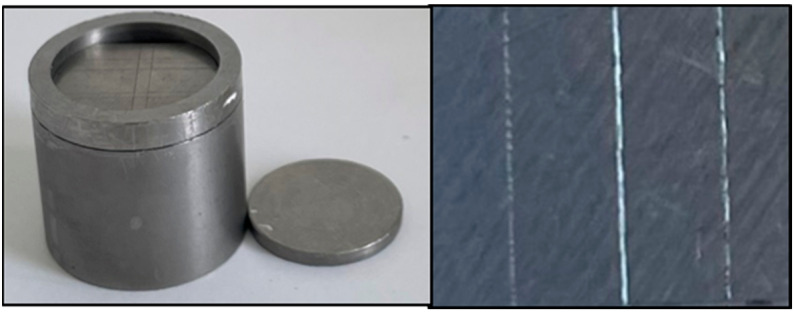
Detail reproduction metal mold with 20, 50, and 75 μm depth engraved lines.

Surface detail reproduction of alginate impression materials was determined according to ISO specification 1563 but with a slight modification of the specified metallic mold to allow measurements of the dimensional accuracy in the X and Y axes [27]. A stainless-steel mold was used, which was engraved with three vertical orientation lines (20, 50, and 75 μm depth and 25 mm length, Figure 5) and two horizontal lines. For detail reproduction assessment, the different alginate groups were prepared and placed inside the mold, and then covered with a metal plate with 1 kg weight on top to simulate the impression-taking process and allow for the outflow of excess material. After gelation, the surface of each specimen (*n* = 6 for each group) was examined using a stereomicroscope (Wild Lecia M8, Heerbrugg, Switzerland) at 10× magnification. Detail reproduction was examined according to the ISO specification number 1563, in which the alginate impression materials had to reproduce the full length of the 50 μm line over the full 25 mm length. A score of one was set to a line that was fully and continuously reproduced, and otherwise, a zero score was given.

**Figure 6:**
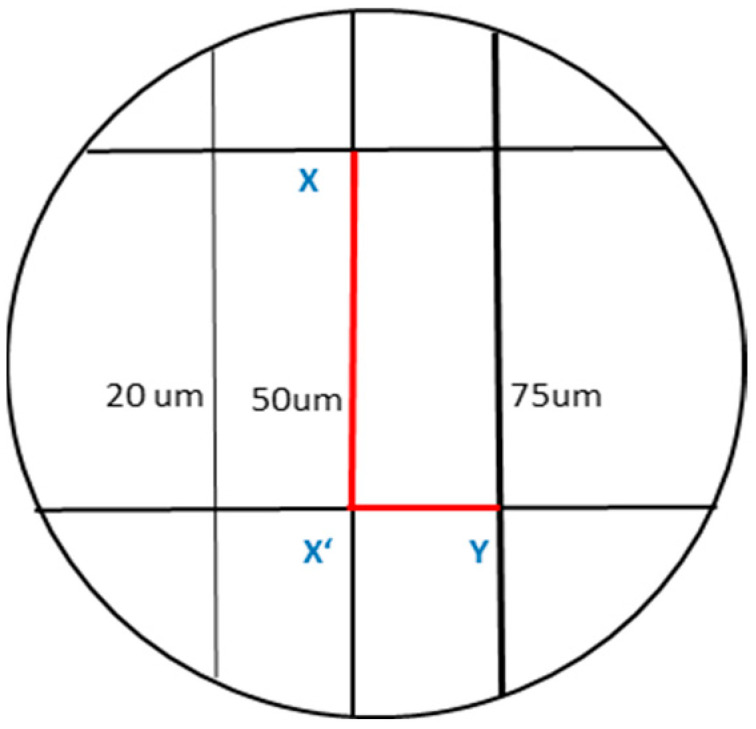
Schematic drawing of the stainless-steel analog indicating the lines measured between reference points X-X’ (Vertical dimension) and X’-Y (horizontal dimension).

For dimensional change examination, the samples of the detail reproduction test were photographed using the stereomicroscope (Wild Lecia M8, Heerbrugg, Switzerland) combined with a digital camera (Leica DFC 420 C, Leica Mikrosysteme, Wetzlar, Germany) at 18× magnification. The vertical dimension between reference points (X, X′) and horizontal dimensions between X′ and Y in each specimen were measured using Leica LAS AF LITE 4.10.0 software (Figure 6). Dimensional changes were assessed by calculating the difference between reference points of the master model and its replica, shortly after impression making [48]. The value for dimensional change was calculated as an average of three measurements and was recorded to the nearest 0.1 mm.

#### 4.2.2. Tear Strength

**Figure 7:**
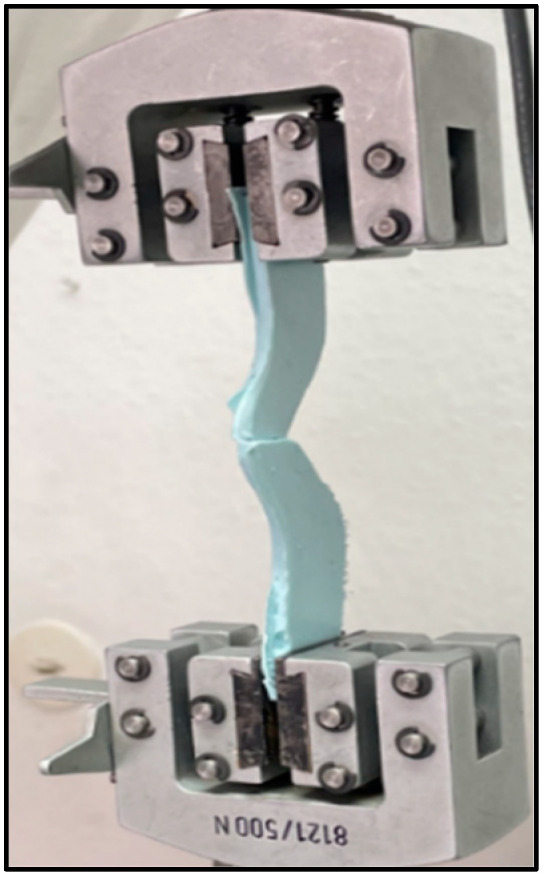
Tear strength specimen clamped in a Zwick testing machine.

A tear strength test was performed according to ISO standard 1563:1990 [27] for irreversible hydrocolloid impression materials. A polymeric mold was printed using a 3D printer (Renkforce RF100, Conrad, Hirschau, Germany) with dimensions of 10 cm length, 2 cm width, and 4 mm thickness at the tearing point (Figure 7). The mold was placed over a glass plate and filled with the freshly mixed alginate prepared for each group. The filled mold was then covered with a second glass plate and a weight of 500 gm was added over the top to ensure uniform alginate thickness. After setting, the specimens were inspected for defects, excess material was trimmed, and the thickness of each sample was recorded using a digital caliper (Mitutoyo GmbH, Neuss, Germany). Each specimen was fixed to the grips of a Zwick materials testing machine (Zwick Zmart Pro, ZwickRoell GmbH & Co. KG, Ulm, Germany) and subjected to tensile load at a crosshead speed of 500 mm/minute until rupture. Tear strength was calculated according to the following equation:

$$Tear\;strength=\frac{Force\;required\;for\;tearing}{Thickness\;of\;sample}$$

#### 4.2.3. Elastic Recovery

**Figure 8:**
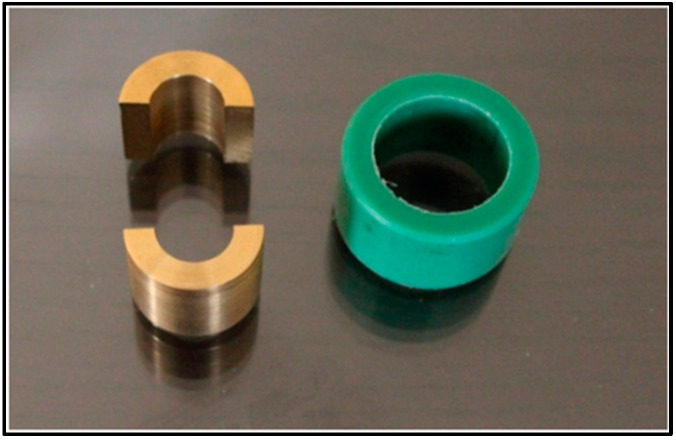
Copper split mold and a plastic fixation ring for elastic recovery testing.

Elastic recovery was evaluated using a split cylindrical mold (20 mm in length and a 12.5 mm interior diameter), surrounded by a fixation ring as recommended by ISO 1563:1990 (Figure 8) [26]. Alginate powder was mixed with the designated liquid for each group and allowed to set inside the mold (*n* = 10). After the setting time recommended by the manufacturer, the samples were inspected and measured using a digital caliper by the same practitioner for standardization. A Zwick material testing machine (Zwick Zmart Pro, Zwick Roell GmbH & Co. KG, Ulm, Germany) was used to gradually deform each sample by 20% of the original length (L) for 5 s, and then the samples were gradually unloaded to allow for recovery from the deformation. After 40 s as a recovery time, samples were measured again and the recovery from deformation was calculated as percentages by using the following formula:

$$Elastic\;recovery=\left(\frac{\Delta L}{L}-1\right)\times 100$$

where L is the original length and ΔL is the length after deformation.

#### 4.2.4. Statistical Analysis

The Shapiro–Wilk test was used to check normality, and results were presented as mean and standard deviation (SD). All quantitative variables showed parametric distribution; therefore, one-way analysis of variance (ANOVA) was used for comparison between the groups. Tukey’s post hoc test was used for pairwise comparison between the groups when the ANOVA test was significant. The significance level was set at *p* ≤ 0.05. Statistical analysis was performed using Minitab 17.1.0 for Microsoft Windows.

## Data Availability

The datasets used and/or analyzed during the current study are available from the corresponding author upon reasonable request.
